# Routine programmatic data show a positive population-level impact of HIV self-testing: the case of Côte d’Ivoire and implications for implementation

**DOI:** 10.1097/QAD.0000000000003328

**Published:** 2022-08-03

**Authors:** Arlette Simo Fotso, Cheryl Johnson, Anthony Vautier, Konan Blaise Kouamé, Papa Moussa Diop, Romain Silhol, Mathieu Maheu-Giroux, Marie-Claude Boily, Nicolas Rouveau, Clémence Doumenc-Aïdara, Rachel Baggaley, Eboi Ehui, Joseph Larmarange

**Affiliations:** a Centre Population & Développement, Université Paris-Cité, IRD, Inserm, Paris; b French Institute for Demographic Studies, INED, Aubervilliers, France; c World Health Organization, Geneva, Switzerland; d Solidarité Thérapeutique et Initiatives pour la Santé, Solthis, Dakar, Sénégal; e Programme National de Lutte contre le Sida, Abidjan, Côte d’Ivoire; f Medical Research Council Centre for Global Infectious Disease Analysis, School of Public Health, Imperial College London, London, United Kingdom; g Department of Epidemiology and Biostatistics, School of Population and Global Health, McGill University, Montréal, QC, Canada; h Full composition of the ATLAS Team provided in the Supplementary Materials.

**Keywords:** antiretroviral treatment, diagnosis, female sex workers, HIV self-testing, implementation, key populations, men who have sex with men, testing, triangulation of programmatic data

## Abstract

We estimate the effects of ATLAS's HIV self-testing (HIVST) kit distribution on conventional HIV testing, diagnoses, and antiretroviral treatment (ART) initiations in Côte d’Ivoire.

**Design:**

Ecological study using routinely collected HIV testing services program data.

**Methods:**

We used the ATLAS's programmatic data recorded between the third quarter of 2019 and the first quarter of 2021, in addition to data from the President's Emergency Plan for AIDS Relief dashboard. We performed ecological time series regression using linear mixed models. Results are presented per 1000 HIVST kits distributed through ATLAS.

**Results:**

We found a negative but nonsignificant effect of the number of ATLAS’ distributed HIVST kits on conventional testing uptake (−190 conventional tests; 95% confidence interval [CI]: −427 to 37). The relationship between the number of HIVST kits and HIV diagnoses was significant and positive (+8 diagnosis; 95% CI: 0 to 15). No effect was observed on ART initiation (−2 ART initiations; 95% CI: −8 to 5).

**Conclusions:**

ATLAS’ HIVST kit distribution had a positive impact on HIV diagnoses. Despite the negative signal on conventional testing, even if only 20% of distributed kits are used, HIVST would increase access to testing. The methodology used in this paper offers a promising way to leverage routinely collected programmatic data to estimate the effects of HIVST kit distribution in real-world programs.

## Introduction

In 2019, up to 19% of people with HIV (PWH) worldwide were not aware of their HIV status [1]. In Western Africa, this proportion of undiagnosed PWH reached 33% in 2020 [2]. This is well below the *Joint United Nations Programme on HIV/AIDS* (UNAIDS) target to achieve <5% of PWH being undiagnosed by 2025. HIV testing is a crucial element of responses to HIV, as it is the first step to linkage to care and treatment. HIV testing is also key for prevention, as PWH on antiretroviral treatment (ART) and virally suppressed will not transmit HIV to their sexual partners [3].

The World Health Organization (WHO) recommends HIV self-testing (HIVST), which allows individuals to test themselves and learn their results when and how they want [4]. It is an innovative tool that has been demonstrated to be safe, accurate, empowering, and acceptable and to also consistently increase the uptake and frequency of HIV testing across settings and populations [5–12]. It is recommended that a reactive HIVST must be followed by a conventional test to confirm or disprove the results.

In Southern and East Africa, HIVST has been scaled up quickly, catalyzed by the Unitaid-funded Self-test Africa (STAR) initiative, which was started in 2015 [13]. However, before 2019, HIVST was offered only in West Africa through small-case pilot projects [14]. A medium-scale HIVST program was implemented in Côte d’Ivoire, Mali, and Senegal in 2018, with an effective distribution of kits through the ATLAS project funded by Unitaid and implemented by a consortium led by *Solidarité thérapeutique et initiatives pour la santé* (Solthis) and the French Research Institute for Sustainable Development (IRD) since 2019 [15]. From 2019 to 2022, together with national programs, ATLAS planned to deliver 400 000 HIVST kits (214 000 in Côte d’Ivoire). The ATLAS program had set a target for 90% of HIVST implementation to reach key populations (KPs) and their sexual partners, peers and clients. In West Africa, the epidemic remains concentrated in KPs, such as female sex workers (FSW), men who have sex with men (MSM), and people who use drugs (PWUD); and is partly drawn by some vulnerable group such as clients of FSW and their non-FSW female partners [16–18]. The remaining priority populations of the ATLAS HIVST implementation were people with sexually transmitted infections (STIs), their partners and partners of people living with HIV.

ATLAS activities rely on both primary and secondary distribution channels. With primary distribution, HIVST kits are distributed by peer educators and frontline healthcare workers to primary contacts − MSM, PWH, STI patients, FSW, and PWUD − for their personal use. For secondary distribution, primary contacts are invited to redistribute HIVST kits to their peers, partners, clients and relatives. These secondary contacts are often members of key and vulnerable populations who often do not have easy access to the health system, including sexual partners of PWH or members of KPs. This specificity of HIVST kit distribution implies that HIVST beneficiaries (end-users) are not limited to primary contacts. ATLAS's program results have shown that HIVST can reduce stigma; preserve anonymity and confidentiality; reach KPs that do not access testing via other testing approaches; save opportunity costs for users and providers; empower users with autonomy and responsibility; and is noninvasive and easy to use [19–22].

Several programs have developed methods to assess the use of HIVST and test results, such as supervision by health workers, the return of used kits, messages or phone call reminders to return used samples, the electronic transmission of photographs of test results, or the use of Bluetooth sensors [23]. However, such tracking can be costly and counterproductive by limiting the use and distribution of HIVST and is not in line with the philosophy of HIVST, where users can anonymously decide when and where they are tested and if and to whom they want to report their results. The systematic tracking of HIVST through secondary distribution is logistically challenging and can also hinder the secondary distribution of HIVST, as primary contacts can be reluctant to redistribute an HIVST kit if they need to collect contact information. It could also be challenging for tracking HIVST at a large scale due to the logistics it might involve. To preserve the anonymity and confidentiality of those using HIVST and not impede the use of HIVST, ATLAS decided not to systematically track distributed HIVST kits. Nevertheless, HIVST users can still, if they wish, obtain additional support by calling a peer educator or a national HIV hotline.

Without systematic and direct feedback regarding the use and results of HIVST and linkage to confirmatory testing and ART, it is challenging to estimate the population-level impacts of HIVST distribution [24]. In this paper, we aimed to circumvent this problem by using routinely collected programmatic data to estimate the effects of ATLAS's HIVST distribution on conventional HIV testing (i.e., self-testing excluded), HIV diagnoses, and ART initiations in Côte d’Ivoire.

## Methods

### Data sources

ATLAS HIVST distribution in Côte d’Ivoire started during the third quarter of 2019 (Q3 2019) among individuals aged 16 years or older (minimum legal age for HIV testing without parental consent). All ATLAS implementing partners reported the number of HIVST kits distributed through ATLAS monthly by distribution site, delivery channel, age group and sex of primary contacts. Data were aggregated per health district and quarter of the year. In 2020, Côte d’Ivoire was divided into 33 health regions and 113 health districts.

Routine programmatic data for adults over 15 years of age were obtained from the *President's Emergency Plan for AIDS Relief* (PEPFAR) open-access public repository (https://data.pepfar.gov/). PEPFAR is the principal donor to the national AIDS program in Côte d’Ivoire. It collects programmatic data in the health districts where it intervenes, including the number of HIVST kits distributed through PEPFAR-funded activities; conventional testing (i.e. the number of ‘*individuals tested for HIV who received results*’); HIV diagnoses (i.e. the number of ‘*individuals who newly tested positive for HIV*’); and ART initiations (i.e. the number of ‘*people newly enrolled to receive ART*’).

For this study, we used these two sources of quarterly data aggregated at the health district level − harmonized according to the 2020 subdivision − from Q3 2019 to Q1 2021. Over this period, the PEPFAR data were only available for 78/113 (69%) Ivorian health districts. Only these districts were included in the analysis.

This study does not raise any ethical concern, as the data used are aggregated and completely anonymized. A secondary analysis of the ATLAS programmatic data is included in the associated research protocol approved by the WHO Ethical Research Committee, the National Ethics Committee for Life Sciences and Health of Côte d’Ivoire, the Ethics Committee of the Faculty of Medicine and Pharmacy of the University of Bamako, and the National Ethics Committee for Health Research of Senegal.

### Modeling strategy

Our analysis considered three outcomes: the number of conventional HIV tests, HIV diagnoses, and ART initiations. These three outcomes were obtained directly from the PEPFAR datasets.

We used ecological time series regression to model the linear effect of the number of HIVST kits distributed through ATLAS for each outcome [25]. We first used linear mixed models with district-level random effects, as presented in Equation (1):


(1) $$E[y_{i,t}]=\beta_{1}\cdot HIVST_{i,t}+\beta_{2}\cdot T_{t}+d_{i}$$

where *y*_*i,t*_ is the outcome of district *i* at time *t*. *HIVST* is the number of HIVST kits distributed through ATLAS for district *i* at time *t*. *β*_1_ represents the effect of the latter variable on the outcomes. *T*_*t*_ is calendar time, which captures conjectural effects in vector *β*_2_. Conjectural effects were modeled as a categorical variable of the quarter of the year to account for any nonlinear or non-polynomial trend [26]. Modeling time as a categorical variable is also equivalent to running a time fixed-effects model, which allows controlling for variables that are constant across districts but vary over time such as shocks that might occur over the time. *d*_*i*_ is the district-specific random effect. District-level random effects were used to account for autocorrelation due to multiple observations and to produce standard errors adjusted for clustering.

Then, contextual effects were also taken into account by introducing the categorical variable of health regions (*R*_*i*_), and related vector of coefficients β_3_, in the model defined by Equation (2):


(2) $$E[y_{i,t}]=\beta_{1}\cdot HIVST_{i,t}+\beta_{2}\cdot T_{t}+\beta_{3}\cdot R_{i}+d_{i}$$

For each outcome, both Models (1) and (2) were run and results are presented in Supplemental Digital Content.

ATLAS activities were implemented in nine of the 78 districts covered by the PEPFAR dataset. We performed a sensitivity analysis by restricting the sample to these nine health districts. In addition, we assessed the robustness of our results by using cubic splines instead of a categorical variable for modeling time and compared the AIC (Akaike information criterion) of the models.

All analyses were performed in R (version 4.0.3) using the ‘*lme4*’ package for statistical models [27].

## Results

### Descriptive statistics

**Fig. 1:**
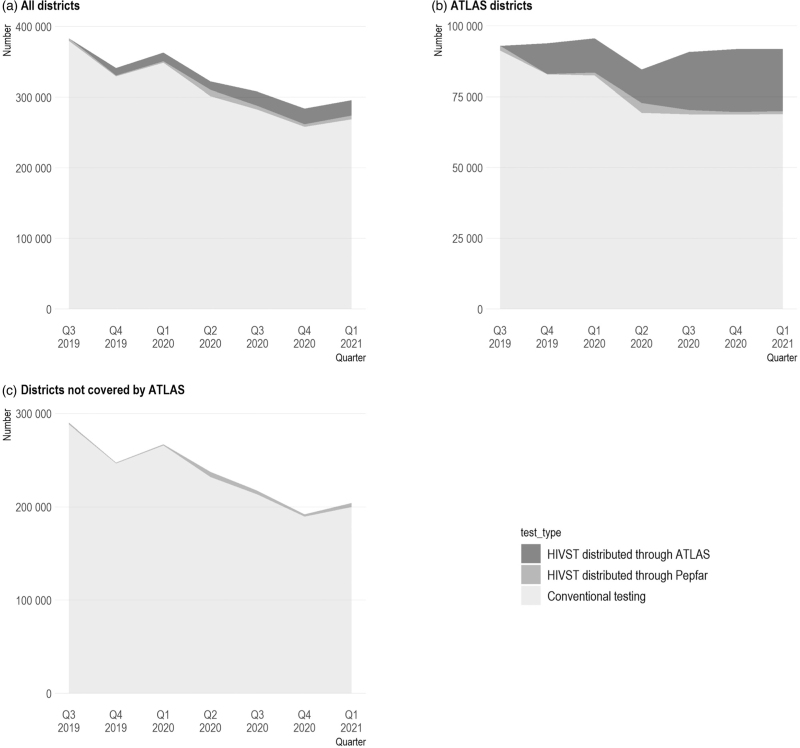
Number of conventional testing and HIV self-testing (HIVST) kits distributed through PEPFAR and ATLAS from Q3 2019 to Q1 2021 in (a) all 78 health districts monitored by PEPFAR in Côte d’Ivoire, (b) the nine ATLAS districts only, and (c) the 69 districts not covered by ATLAS.

In the 78 health districts monitored by PEPFAR, between Q3 2019 and Q1 2021, 30 781 HIVTS kits were distributed through PEPFAR, and 99 353 HIVST kits were distributed through ATLAS, compared with 2 167 828 conventional tests performed over the same period (Table 1). High disparities in terms of volume were observed between districts, with a minimum of 1832 conventional tests and a maximum of 139 214 (median of 13 348). The nine districts − out of 78 where ATLAS activities were implemented − accounted for one quarter (532 287/2 167 828) of conventional tests. Important variations were observed in terms of HIV diagnoses, and ART initiations across districts. In the 78 districts included in the analysis, conventional testing decreased between Q3 2019 and Q1 2021, from 379 554 individuals tested for HIV who received their results in Q3 2019 to 268 807 in Q1 2021 (Fig. 1a). In the 69 districts that were not covered by ATLAS (Fig. 1c), HIVST kits distributed through PEPFAR remained limited and largely insufficient to compensate for the reduction in conventional testing; only 13% of the tests in these districts were HIVST kits. In the nine ATLAS districts, HIVST kit distribution − mainly through ATLAS, but also partially through PEPFAR − has increased continuously since Q3 2019 (Fig. 1b), with a slow-down in Q2 2020, when governmental coronavirus disease 2019 (COVID-19) measures were introduced. Overall, the shock caused by the COVID-19 pandemic is observed in Q2 2020.

**Table 1:** District characteristics and activities between Q3 2019 and Q1 2021 in 78 health districts monitored by PEPFAR in Côte d’Ivoire.

Variable	All districts, *N* = 78	ATLAS districts, *N* = 9	Districts not covered by ATLAS, *N* = 69
Conventional testing			
Sum	2 167 828	532 287	1 635 541
Median	19 348	57 037	18 162
Range	1832–139 214	13 914–139 214	1832–78 847
HIV diagnoses			
Sum	60 716	16 143	44 573
Median	484	1465	467
Range	33–3862	251–3862	33–2749
ART initiations			
Sum	54 354	13 846	40 508
Median	430	1414	422
Range	33–3068	216–3068	33–2274
HIVST distributed through ATLAS			
Sum	99 353	99 353	0
Median	0	10 968	0
Range	0–23 472	1364–23 472	0–0
HIVST distributed through PEPFAR			
Sum	30 781	9881	20 900
Median	168	735	100
Range	0–2536	102–2536	0–1881

**Fig. 2:**
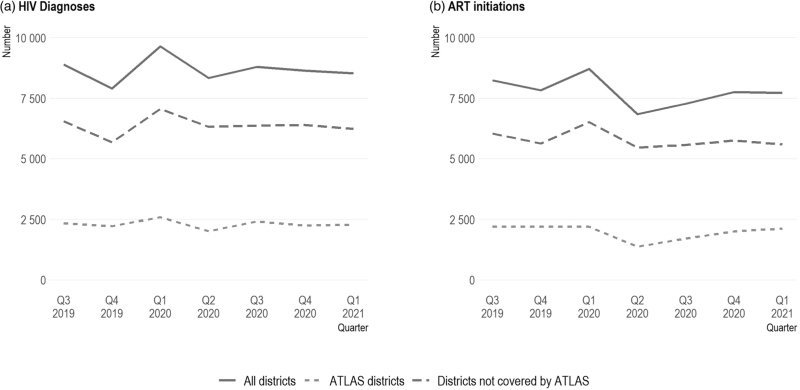
Number of HIV diagnoses and ART initiations in the 78 health districts monitored by PEPFAR in Côte d’Ivoire (Q3 2019 to Q1 2021).

HIV diagnoses and ART initiations remained relatively stable over time (Figs. 2a and b), with a catch-up effect observed in Q1 2020 after a slowdown in Q4 2019. Trends were similar in the ATLAS districts and the districts not covered by ATLAS.

### Regression results

When adjusting for time and region (first columns of Table 2), the estimated effect of ATLAS HIVST kit distribution shows a nonsignificant negative signal on conventional testing, with a decline of −195 [95% confidence interval (CI): −427 to 38, *P* = 0.10] conventional tests for every 1000 additional HIVST kits distributed by ATLAS. HIVST kit distribution was positively associated with HIV diagnoses: +8 diagnoses [95% CI: 0 to 15, *P* = 0.04] per 1000 additional HIVST kits distributed. No association between HSVST kit distribution and ART initiations was observed: −2 [95% CI: −8 to 5, *P* = 0.66]. Similar results were observed when adjusting only for time regarding the linear effect of the number of HIVST kits distributed through ATLAS on the different outcomes (first columns of Table A1, Supplemental Digital Content, Table A2, Supplemental Digital Content and Table A3, Supplemental Digital Content). Full regression tables are presented in the second columns of Table A1, Supplemental Digital Content, Table A2, Supplemental Digital Content and Table A3, Supplemental Digital Content.

**Table 2:** Linear effect of the number of HIVST kits distributed through ATLAS on access to HIV testing, conventional tests, diagnoses and ART initiations in the health districts monitored by PEPFAR in Côte d’Ivoire (Q3 2019 to Q1 2021).

Outcome	All districts			ATLAS districts		
	Coef.	95% CI^a^	*P*-value	Coef.	95% CI^a^	*P*-value
Conventional testing	−195	−427 to 38	0.10	112	−527 to 750	0.73
HIV diagnoses	8	0 to 15	0.04	14	−10 to 38	0.25
ART initiations	−2	−8 to 5	0.66	5	−14 to 25	0.57

a CI, confidence interval; Coef., coefficient. For the three outcomes, only the regression coefficients of the number of HIVST kits distributed through ATLAS are presented. Coefficients represent the unit change (e.g., conventional tests, diagnoses, ART initiations) per 1000 HIVST test kits distributed through ATLAS. For the full regression table, see the Supplementary Material (Table A1, Supplemental Digital Content through Table A3).

When restricting the analyses to the nine ATLAS districts, the estimated magnitudes of association were larger, though not statistically significant (second columns of Table 2). The sensitivity analyses modeling time with cubic splines instead of categorical variables showed very similar results (Table A4, Supplemental Digital Content). However, the effect on HIV diagnoses was no longer significant. A comparison of the AIC values of the models indicated that the models with categorical variables fit the time series better (Table A5, Supplemental Digital Content).

## Discussion

Using routinely collected programmatic data aggregated quarterly at the health district level, our analyses showed a significant positive effect of HIVST kits distributed through ATLAS on HIV diagnoses. Our results suggested a possible negative signal, albeit not statistically significant, on conventional testing, and no observable effect on the most distal outcome of ART initiations.

HIVST could lead to some substitution effects if HIVST kits are used by individuals who would have undergone a conventional HIV test in their absence, as observed in other studies [5,28]. Such effects may be concerning for policy-makers, as gains in HIV testing coverage due to HIVST distribution may result in a reduction in the number of conventional HIV tests. Our results did show a negative effect of HIVST kit distribution on conventional testing (−195 conventional tests for every 1000 distributed HIVST) but the uncertainty was large and we cannot rule out the absence of substitution effects. However, even if this substitution effect was significant and that only 20% of distributed HIVST kits were used, the net impact on testing uptake would be positive: 200 additional HIVST performed for every 1000 distributed HIVST would reduce the number of conventional tests by 195 (net impact of +5). The existing literature reports utilization rate of HIVST that could reach up to 80% [29], suggesting our 20% utilization rate assumption is conservative. Moreover, the descriptive data showed that a decrease in conventional testing occurred in all districts, including those not covered by ATLAS activities, this is linked to the fact that PEPFAR's testing strategies are revised annually and favoring more targeted approaches [30]. Our results suggest that ATLAS HIVST distribution help maintain access to HIV testing in its implementation districts despite the slowdown observed in Q2 2020 when governmental COVID-19 measures were introduced. In fact, a main takeaway from the ATLAS project is that HIVST distribution activities among KPs can be easily adapted, including in the context of the COVID-19 pandemic [21].

Due to its confidential nature, HIVST could overcome several structural barriers for HIV diagnoses – such as stigma and opportunity costs – and create new approaches to reach first-time testers and boost HIV retesting for KPs, therefore improving access to HIV testing overall. These results are in line with previous studies among KPs in East and South Africa [5,28,31].

If HIVST is appropriately used as a triage test and individuals with reactive self-tests are linked for confirmatory testing, HIVST distribution activities should lead to a higher number of positive tests in conventional testing. Several actors have expressed concern that HIVST could have a negative impact on new diagnoses [32]. In fact, at the beginning of the ATLAS project, key stakeholders, though recognizing the potential of HIVST to reach first-time testers, expressed some doubts regarding users’ ability to accept a reactive test result. There were concerns that individuals with reactive tests would not seek confirmatory testing, which would limit the number of new diagnosed observed at health facilities [19]. Our results did not show any deleterious effect on HIV diagnoses but rather showed a significant positive effect. For the 99 353 HIVST distributed through ATLAS over the period Q3 2019 through Q1 2021, this could translate to 795 additional diagnoses. This is in line with some other studies, such as that by MacGowan *et al.*[33], who found that the number of HIV infections detected in their HIVST arm was higher compared to the control arm in a randomized trial conducted among MSM.

Our model did not observe any effect of ATLAS HIVST kit distribution on ART initiations. The estimated effect was negative when all 78 districts were included and positive when the analysis was limited to the nine ATLAS districts. The analysis with nine districts could suffer from a lack of power. However, in all instances, the effect size estimate was small. The PEPFAR datasets are not exhaustive for Côte d’Ivoire and cover only 78 out of the 113 health districts at the national level and 9 of the 12 ATLAS districts.

Using aggregated data rather than individual data implies a lower number of observation points and therefore lower statistical power, although these data allow us to make population-level estimates. Aggregated data is subject to ecological bias and statistical relationships must be interpreted with caution. In addition, it is not possible to completely rule out any ‘contamination’ effect, as individuals living on district borders could perform conventional testing in the neighboring district. However, we could assume that population movement at boundaries could happen in both directions, thus compensating for each other, or expect the observed effect to have been even stronger without a ‘contamination’ effect. The collected data did not allow us to distinguish between confirmatory tests following HIVST and classic conventional tests, but as the HIV prevalence is relatively low in the country, the former number might not be important. Finally, HIVST kits distributed through UNICEF or the Global Fund to fight AIDS, tuberculosis and malaria at district level and by yearly quarter are not available. Nevertheless, the volumes of tests distributed by these programs were very low, 6879 and 1373 kits, respectively, by 2020, representing less than 7% of all HIVST kits distributed in the country.

A strength of this study is that it specifically used only indicators that had already been routinely collected by countries, which means that the method could be easily replicated in other contexts and used by other countries to monitor the impact of their HIVST activities without any additional cost. Our analysis did not rely on any systematic tracking system or data collection process, which can be expensive and complex and are counter to the rationale for HIVST.

A core component of the ATLAS HIVST strategy in West Africa is the secondary distribution of HIVST kits, primarily distributed through activities targeting individuals in KPs, in particular FSW and MSM. It is therefore expected that many HIVST users would not self-identify as being in a KP and that those with a reactive test would not link to partner community facilities serving KPs for confirmatory testing but rather to more general public or private facilities, making it difficult to link specific records with the distribution of HIVST kits. In addition, records of prior HIVST kit use at health facilities are expected to be underestimated, as recognizing such use would mean the individual was a member of and/or in a network of a KP. By using data aggregated at the district level and covering all testing facilities, confirmatory tests prompted by reactive HIVST results are considered, regardless of where they occurred. By allowing programs to shift from systematic tracking for evaluation, such indirect evaluation would help to focus on and increase access to HIV testing for hard-to-reach populations and first-time testers and allow large-scale secondary distribution implementation.

### Conclusion

To the best of our knowledge, this represents the first study estimating the impact of HIVST kit distribution at the population level in West Africa. Our results highlight that a social network-based HIVST distribution strategy, focusing on key population members as primary contacts but aiming to reach their partners and social contacts, does have a positive impact on diagnoses that is observable at the population level.

Such evaluation is pragmatic and could be performed with routinely collected programmatic indicators. The WHO recommends reporting on the number of HIVST kits distributed and estimating HIVST access and use through population-based surveys. Countries are burdened with multiple HIV reporting systems and numerous indicators. It could therefore be of considerable benefit to monitor the impact of self-testing through current data systems without introducing new indicators and further data collection. This method of triangulating available data provides further information on the population-level impacts of HIV self-testing to guide program use.

## Acknowledgements

Author contributions: A.S.F. and J.L. conceptualized the paper. A.S.F. and P.M.D. curated the data. A.S.F. performed the formal analysis, wrote the initial computer programs and wrote the first draft of the paper, while J.L. supervised, validated and ensured the reproducibility of the results and was in charge of the project administration. A.S.F. and J.L. defined the methodology and visualization adopted for the paper. A.S.F., P.M.D. and A.V. provided the resources used for the research. J.L., A.V. and C.D. secured funding for the project. A.S.F., K.B.K., E.E., A.V. and P.M.D. were involved in the investigation. All authors wrote, reviewed and edited the paper. The findings and conclusions in this paper are those of the authors and do not necessarily represent the official position of the funding agencies.

Funding: The ATLAS Project was funded by Unitaid, Grant number 2018-23-ATLAS.

Ethics statement: This study does not raise any ethical concern, as the data used are aggregated and thus completely anonymized.

A secondary analysis of the ATLAS programmatic data is included in the associated research protocol available at https://atlas.solthis.org/en/research/. This protocol (version 2.1, 5 August 2019) was approved by the WHO Ethical Research Committee (7 August 2019, Reference: ERC 0003181), the National Ethics Committee for Life Sciences and Health of Côte d’Ivoire (28 May 2019, Reference: 049-19/MSHP/CNESVS-kp), the Ethics Committee of the Faculty of Medicine and Pharmacy of the University of Bamako, Mali (14 August 2019, Reference: 2019/88/CE/FMPOS), and the National Ethics Committee for Health Research of Senegal (26 July 2019, Protocol SEN19/32).

Publication history: This manuscript was previously posted to medRxiv: doi: https://doi.org/10.1101/2022.02.08.22270670

### Conflicts of interest

C.J. declares that WHO receives grants to support activities on HIV testing including self-testing from USAID, Unitaid and the Bill and Melinda Gates Foundation

All the other authors declare no conflicts of interest.

## Supplementary Material

Supplemental Digital Content
