# Supplementary material for: Routine programmatic data show a positive population-level impact of HIV self-testing: the case of Côte d’Ivoire and implications for implementation
Source: AIDS. 2022 Aug 3;36(13):1871–9. doi: 10.1097/QAD.0000000000003328 (PMC9594126; doi:10.1097/QAD.0000000000003328)
Supplement: Supplemental Digital Content [file aids-36-1871-s001.docx]

# Additional files

**Table of contents**

[Additional files 1](#_Toc107391867)

[Supplementary tables 1](#_Toc107391868)

[Composition of the ATLAS Team 8](#_Toc107391869)

[Data and R code 13](#_Toc107391870)

## Supplementary tables

**Table A1. Linear effect of the number of HIVST kits distributed through ATLAS on HIV conventional testing in the health districts monitored by PEPFAR in Côte d’Ivoire (Q3 2019 to Q1 2021)**

|  | Adjusted for time | | | Adjusted for time and region | | | ATLAS districts only Adjusted for time and region | | |
| --- | --- | --- | --- | --- | --- | --- | --- | --- | --- |
| Characteristics | Coef. | 95% CI^1^ | p-value | Coef. | 95% CI^1^ | p-value | Coef. | 95% CI^1^ | p-value |
| Number of HIVST distributed through ATLAS | -133 | -365 to 99 | 0·26 | -195 | -427 to 38 | 0·10 | 112 | -527 to 750 | 0·73 |
| Quarter year |  |  |  |  |  |  |  |  |  |
| 2019 Q3 | — | — |  | — | — |  | — | — |  |
| 2019 Q4 | -624 | -924 to -323 | 0·00 | -615 | -916 to -315 | 0·00 | -1 065 | -2 762 to 632 | 0·21 |
| 2020 Q1 | -378 | -679 to -76 | 0·01 | -368 | -669 to -67 | 0·02 | -1 128 | -2 868 to 613 | 0·20 |
| 2020 Q2 | -984 | -1 284 to -683 | 0·00 | -974 | -1 275 to -674 | 0·00 | -2 583 | -4 310 to -855 | 0·00 |
| 2020 Q3 | -1 214 | -1 519 to -909 | 0·00 | -1 198 | -1 503 to -893 | 0·00 | -2 751 | -4 845 to -657 | 0·01 |
| 2020 Q4 | -1 519 | -1 825 to -1 213 | 0·00 | -1 502 | -1 808 to -1 196 | 0·00 | -2 785 | -4 969 to -602 | 0·01 |
| 2021 Q1 | -1 382 | -1 688 to -1 077 | 0·00 | -1 365 | -1 671 to -1 060 | 0·00 | -2 758 | -4 925 to -592 | 0·01 |
| Health region |  |  |  |  |  |  |  |  |  |
| Abidjan 1-Grands Ponts | — | — |  | — | — |  | — | — |  |
| Abidjan 2 |  |  |  | 6 174 | 2 781 to 9 566 | 0·00 | 4 728 | -6 271 to 15 727 | 0·32 |
| Agneby-Tiassa |  |  |  | -2 610 | -6 357 to 1 138 | 0·17 |  |  |  |
| Bagoue |  |  |  | -4 754 | -9 129 to -378 | 0·03 |  |  |  |
| Belier |  |  |  | -2 746 | -6 494 to 1 001 | 0·15 |  |  |  |
| Bere |  |  |  | -4 899 | -8 647 to -1 152 | 0·01 |  |  |  |
| Cavally |  |  |  | -4 773 | -9 149 to -397 | 0·03 |  |  |  |
| Gbeke |  |  |  | -2 686 | -5 676 to 304 | 0·08 |  |  |  |
| Gbokle-Nawa-San Pedro |  |  |  | 1 170 | -2 226 to 4 566 | 0·49 | -1 295 | -11 337 to 8 747 | 0·75 |
| Goh |  |  |  | -2 453 | -6 200 to 1 295 | 0·19 |  |  |  |
| Gontougo |  |  |  | -3 862 | -7 018 to -706 | 0·02 |  |  |  |
| Guemon |  |  |  | -3 646 | -7 394 to 101 | 0·06 |  |  |  |
| Hambol |  |  |  | -3 646 | -9 516 to 2 224 | 0·22 |  |  |  |
| Haut-Sassandra |  |  |  | -1 238 | -4 628 to 2 152 | 0·47 |  |  |  |
| Iffou |  |  |  | -3 611 | -9 481 to 2 259 | 0·22 |  |  |  |
| Indenie-Djuablin |  |  |  | -2 048 | -6 423 to 2 328 | 0·35 |  |  |  |
| Loh-Djiboua |  |  |  | 357 | -5 513 to 6 227 | 0·90 |  |  |  |
| Marahoue |  |  |  | -1 881 | -5 629 to 1 866 | 0·32 |  |  |  |
| Me |  |  |  | -3 386 | -6 775 to 4 | 0·05 | -5 687 | -16 677 to 5 303 | 0·24 |
| Moronou |  |  |  | -4 334 | -8 082 to -587 | 0·02 |  |  |  |
| N'zi |  |  |  | -5 070 | -8 818 to -1 323 | 0·01 |  |  |  |
| Poro |  |  |  | -3 326 | -7 074 to 421 | 0·08 |  |  |  |
| Tchologo |  |  |  | -5 063 | -9 438 to -687 | 0·02 |  |  |  |
| Tonkpi |  |  |  | -3 154 | -6 902 to 593 | 0·10 |  |  |  |
| Worodougou |  |  |  | -3 311 | -7 687 to 1 065 | 0·14 |  |  |  |
| ^1^CI = Confidence Interval. Coef.= coefficient. Coefficients represent the unit change (e.g., conventional tests, diagnoses, ART initiations) per 1,000 HIVST test kits distributed through ATLAS. Time is modeled as a categorical variable. | | | | | | | | | |

Bbbb

Table A24. Linear effect of the number of HIVST kits distributed through ATLAS on HIV diagnoses in the 78 health districts monitored by PEPFAR in Côte d’Ivoire (Q3 2019 to Q1 2021)

ccc

**Table A2. Linear effect of the number of HIVST kits distributed through ATLAS on HIV diagnoses in the health districts monitored by PEPFAR in Côte d’Ivoire (Q3 2019 to Q1 2021)**

|  | Adjusted for time | | | Adjusted for time and region | | | ATLAS districts only  Adjusted for time and region | | |
| --- | --- | --- | --- | --- | --- | --- | --- | --- | --- |
| Characteristics | Coef. | 95% CI^1^ | p-value | Coef. | 95% CI^1^ | p-value | Coef. | 95% CI^1^ | p-value |
| Number of HIVST distributed through ATLAS | 9 | 1 to 16 | 0·02 | 8 | 0 to 15 | 0·04 | 14 | -10 to 38 | 0·25 |
| Quarter year |  |  |  |  |  |  |  |  |  |
| 2019 Q3 | — | — |  | — | — |  | — | — |  |
| 2019 Q4 | -14 | -24 to -4 | 0·00 | -14 | -23 to -4 | 0·00 | -31 | -99 to 36 | 0·36 |
| 2020 Q1 | 8 | -1 to 18 | 0·09 | 8 | -1 to 18 | 0·09 | 8 | -62 to 77 | 0·82 |
| 2020 Q2 | -9 | -18 to 1 | 0·08 | -8 | -18 to 1 | 0·09 | -56 | -124 to 13 | 0·11 |
| 2020 Q3 | -4 | -13 to 6 | 0·46 | -3 | -13 to 6 | 0·51 | -24 | -106 to 58 | 0·56 |
| 2020 Q4 | -6 | -16 to 4 | 0·24 | -5 | -15 to 4 | 0·27 | -46 | -132 to 39 | 0·28 |
| 2021 Q1 | -7 | -17 to 3 | 0·15 | -7 | -17 to 3 | 0·17 | -41 | -126 to 43 | 0·33 |
| Health region |  |  |  |  |  |  |  |  |  |
| Abidjan 1-Grands Ponts | — | — |  | — | — |  | — | — |  |
| Abidjan 2 |  |  |  | 210 | 101 to 319 | 0·00 | 84 | -96 to 264 | 0·28 |
| Agneby-Tiassa |  |  |  | -128 | -249 to -8 | 0·04 |  |  |  |
| Bagoue |  |  |  | -161 | -301 to -20 | 0·03 |  |  |  |
| Belier |  |  |  | -116 | -236 to 5 | 0·06 |  |  |  |
| Bere |  |  |  | -167 | -288 to -47 | 0·01 |  |  |  |
| Cavally |  |  |  | -142 | -283 to -2 | 0·05 |  |  |  |
| Gbeke |  |  |  | -109 | -205 to -13 | 0·03 |  |  |  |
| Gbokle-Nawa-San Pedro |  |  |  | -79 | -188 to 30 | 0·15 | -239 | -404 to -75 | 0·01 |
| Goh |  |  |  | -85 | -206 to 35 | 0·16 |  |  |  |
| Gontougo |  |  |  | -123 | -224 to -22 | 0·02 |  |  |  |
| Guemon |  |  |  | -126 | -247 to -6 | 0·04 |  |  |  |
| Hambol |  |  |  | -132 | -321 to 56 | 0·17 |  |  |  |
| Haut-Sassandra |  |  |  | -79 | -188 to 29 | 0·15 |  |  |  |
| Iffou |  |  |  | -145 | -333 to 44 | 0·13 |  |  |  |
| Indenie-Djuablin |  |  |  | -56 | -196 to 85 | 0·43 |  |  |  |
| Loh-Djiboua |  |  |  | -40 | -228 to 149 | 0·68 |  |  |  |
| Marahoue |  |  |  | -98 | -218 to 23 | 0·11 |  |  |  |
| Me |  |  |  | -156 | -265 to -47 | 0·01 | -317 | -497 to -137 | 0·01 |
| Moronou |  |  |  | -162 | -283 to -42 | 0·01 |  |  |  |
| N'zi |  |  |  | -181 | -301 to -61 | 0·00 |  |  |  |
| Poro |  |  |  | -117 | -237 to 4 | 0·06 |  |  |  |
| Tchologo |  |  |  | -169 | -310 to -29 | 0·02 |  |  |  |
| Tonkpi |  |  |  | -81 | -202 to 39 | 0·18 |  |  |  |
| Worodougou |  |  |  | -150 | -290 to -9 | 0·04 |  |  |  |
| ^1^CI = Confidence Interval. Coef.= coefficient. Coefficients represent the unit change (e.g., conventional tests, diagnoses, ART initiations) per 1,000 HIVST test kits distributed through ATLAS. Time is modeled as a categorical variable. | | | | | | | | | |

**Table A3. Linear effect of the number of HIVST kits distributed through ATLAS on ART initiations in the health** **districts monitored by PEPFAR in Côte d’Ivoire (Q3 2019 to Q1 2021)**

|  | Adjusted for time | | | Adjusted for time and region | | | ATLAS districts only Adjusted for time and region | | |
| --- | --- | --- | --- | --- | --- | --- | --- | --- | --- |
| Outcome | Coef. | 95% CI^1^ | p-value | Coef. | 95% CI^1^ | p-value | Coef. | 95% CI^1^ | p-value |
| Number of HIVST distributed through ATLAS | 0 | -7 to 6 | 0·90 | -2 | -8 to 5 | 0·66 | 5 | -14 to 25 | 0·57 |
| Quarter year |  |  |  |  |  |  |  |  |  |
| 2019 Q3 | — | — |  | — | — |  | — | — |  |
| 2019 Q4 | -5 | -14 to 3 | 0·24 | -5 | -14 to 4 | 0·25 | -6 | -60 to 47 | 0·81 |
| 2020 Q1 | 6 | -3 to 15 | 0·17 | 6 | -3 to 15 | 0·16 | -8 | -63 to 47 | 0·78 |
| 2020 Q2 | -18 | -27 to -9 | 0·00 | -18 | -26 to -9 | 0·00 | -98 | -153 to -44 | 0·00 |
| 2020 Q3 | -12 | -21 to -3 | 0·01 | -12 | -21 to -3 | 0·01 | -67 | -132 to -2 | 0·04 |
| 2020 Q4 | -6 | -15 to 3 | 0·17 | -6 | -15 to 3 | 0·19 | -35 | -103 to 32 | 0·30 |
| 2021 Q1 | -7 | -15 to 2 | 0·14 | -6 | -15 to 3 | 0·16 | -21 | -89 to 46 | 0·52 |
| Health region |  |  |  |  |  |  |  |  |  |
| Abidjan 1-Grands Ponts | — | — |  | — | — |  | — | — |  |
| Abidjan 2 |  |  |  | 180 | 82 to 277 | 0·00 | 53 | -86 to 193 | 0·36 |
| Agneby-Tiassa |  |  |  | -112 | -220 to -4 | 0·04 |  |  |  |
| Bagoue |  |  |  | -143 | -269 to -17 | 0·03 |  |  |  |
| Belier |  |  |  | -106 | -214 to 2 | 0·06 |  |  |  |
| Bere |  |  |  | -152 | -260 to -44 | 0·01 |  |  |  |
| Cavally |  |  |  | -148 | -274 to -22 | 0·02 |  |  |  |
| Gbeke |  |  |  | -99 | -185 to -13 | 0·02 |  | t |  |
| Gbokle-Nawa-San Pedro |  |  |  | -71 | -169 to 27 | 0·15 | -219 | -346 to -91 | 0·01 |
| Goh |  |  |  | -83 | -191 to 25 | 0·13 |  |  |  |
| Gontougo |  |  |  | -110 | -201 to -19 | 0·02 |  |  |  |
| Guemon |  |  |  | -131 | -239 to -23 | 0·02 |  |  |  |
| Hambol |  |  |  | -121 | -290 to 48 | 0·16 |  |  |  |
| Haut-Sassandra |  |  |  | -64 | -162 to 34 | 0·19 |  |  |  |
| Iffou |  |  |  | -130 | -299 to 39 | 0·13 |  |  |  |
| Indenie-Djuablin |  |  |  | -40 | -166 to 86 | 0·53 |  |  |  |
| Loh-Djiboua |  |  |  | -38 | -207 to 131 | 0·65 |  |  |  |
| Marahoue |  |  |  | -87 | -195 to 21 | 0·11 |  |  |  |
| Me |  |  |  | -143 | -240 to -45 | 0·00 | -294 | -433 to -154 | 0·00 |
| Moronou |  |  |  | -145 | -253 to -37 | 0·01 |  |  |  |
| N'zi |  |  |  | -163 | -271 to -55 | 0·00 |  |  |  |
| Poro |  |  |  | -106 | -214 to 2 | 0·05 |  |  |  |
| Tchologo |  |  |  | -150 | -276 to -24 | 0·02 |  |  |  |
| Tonkpi |  |  |  | -83 | -191 to 25 | 0·13 |  |  |  |
| Worodougou |  |  |  | -127 | -253 to -1 | 0·05 |  |  |  |
| ^1^CI = Confidence Interval. Coef.= coefficient. Coefficients represent the unit change (e.g., conventional tests, diagnoses, ART initiations) per 1,000 HIVST test kits distributed through ATLAS. Time is modeled as a categorical variable. | | | | | | | | | |

**Table A4:** **Effect of the number of HIVST kits distributed by ATLAS on conventional testing, diagnoses and ART initiations, time modeled using a cubic spline**

|  | All districts | | | ATLAS districts | | |
| --- | --- | --- | --- | --- | --- | --- |
| Outcome | Coef. | 95% CI^1^ | p-value | Coef. | 95% CI^1^ | p-value |
| Conventional testing | -211 | -446 to 23 | 0·08 | 132 | -452 to 715 | 0·65 |
| HIV diagnoses | 7 | 0 to 15 | 0·05 | 16 | -7 to 39 | 0·18 |
| ART initiations | -1 | -8 to 6 | 0·78 | 13 | -6 to 32 | 0·17 |
| ^1^CI = Confidence Interval. Coef.= coefficient. Coefficients represent the unit change (e.g., conventional tests, diagnoses, ART initiations) per 1,000 HIVST test kits distributed through ATLAS. | | | | | | |

**Table A5: Akaike information criterion comparison of the models according to trend modeling**

|  | Outcome | | |
| --- | --- | --- | --- |
| Trend modelling | Conventional testing | HIV diagnoses | ART initiations |
| Time indicator (categorical variable) | 8 893·1 | 5 351·2 | 5 250·7 |
| Cubic splines | 8 933·6 | 5 379·7 | 5 279·4 |

Note: AIC was compared for the models adjusted for time and region.

## Composition of the ATLAS Team

| \| ATLAS Research Team \| \| \| --- \| --- \| \| Amani [Elvis Georges](https://www.notion.so/Elvis-Georges-Amani-d7395a112f144d2cb673123bae5c6329) \| Programme PACCI, ANRS Research Site, Treichville University Hospital, Abidjan, Côte d'Ivoire. \| \| [Badiane](https://www.notion.so/K-ba-Badiane-9447b415454a42e396b6fba69e7ee172) Kéba \| Solthis, Sénégal \| \| Bayac Céline \| Solthis, France \| \| [Bekelynck Anne](https://www.notion.so/Bekelynck-Anne-f6587a3b9a3c4ae5b5630907c0c986fa) \| Programme PACCI, ANRS Research Site, Treichville University Hospital, Abidjan, Côte d'Ivoire \| \| B[oily Marie-](https://www.notion.so/Boily-Marie-Claude-54b3fbd890bf46fbbf5b1e862cf604f0)C[laude](https://www.notion.so/Boily-Marie-Claude-54b3fbd890bf46fbbf5b1e862cf604f0) \| Medical Research Council Centre for Global Infectious Disease Analysis, Department of Infectious Disease Epidemiology, Imperial College London, London, United Kingdom \| \| Boye [Sokhna](https://www.notion.so/Sokhna-Boye-be416eba7a824a8c941ae700840b6795) \| Centre Population et Développement, Institut de Recherche pour le Développement, Université de Paris, Inserm, Paris, France \| \| [Breton Guillaume](https://www.notion.so/Breton-Guillaume-16d3581e74dc45c4a5c0ca31a5ac50ea) \| Solthis, Paris, France \| \| [d’Elbée Marc](https://www.notion.so/d-Elb-e-Marc-b776f2ae8ea34685bd43aed0e36872d5) \| Department of Global Health and Development, Faculty of Public Health and Policy, London School of Hygiene and Tropical Medicine, London, UK \| \| D[esclaux Alice](https://www.notion.so/Desclaux-Alice-7241657b7fff4d009ef03c84afd9f97d) \| Institut de Recherche pour le Développement, Transvihmi (UMI 233 IRD, 1175 INSERM, Montpellier University), Montpellier, France/CRCF, Dakar, Sénégal \| \| [Desgrées du Loû Annabel](https://www.notion.so/Desgree-du-Lou-Annabel-ba07e466040e4407b582f88996d29f5b) \| Centre Population et Développement, Institut de Recherche pour le Développement, Université de Paris, Inserm, Paris, France \| \| Diop Papa Moussa \| Solthis, Sénégal \| \| [Doumenc-Aïdara](https://www.notion.so/Cl-mence-Doumenc-A-dara-a71b566697864ffe9a227acca77af5d3) Clémence \| Solthis, Dakar, Sénégal \| \| Ehui Eboi \| Directeur Coordonnateur, PNLS \| \| Graham Medley \| Department of Global Health and Development, Faculty of Public Health and Policy, London School of Hygiene and Tropical Medicine, London, UK \| \| [Jean Kévin](https://www.notion.so/Jean-K-vin-3a288b7aeb8b4d71a3adf60301a95325) \| Laboratoire MESuRS, Conservatoire National des Arts et Métiers, Paris, France \| \| Keita [Abdelaye](https://www.notion.so/Abdelaye-Keita-6c1586ce65a742509d2e5adcacb082a8) \| Institut National de Recherche en Santé Publique, Bamako, Mali \| \| Kouadio [Brou Alexis](https://www.notion.so/Brou-Alexis-Kouadio-f467590129bf470ebcefec47e06cef47) \| Assistant de recherche, Côte d'Ivoire \| \| [Kouassi Kra](https://www.notion.so/Ars-ne-Kouassi-Kra-21bd2bbd428343ba9256254cf9016f44) Arsène \| Centre Population et Développement, Institut de Recherche pour le Développement, Université de Paris, Inserm, Paris, France \| \| [Ky-Zerbo Odette](https://www.notion.so/Ky-zerbo-Odette-fbe49e1a096547ba90bbd1b76181034b) \| TransVIHMI, IRD, Université de Montpellier, INSERM \| \| L[armarange](https://www.notion.so/Larmarange-Joseph-1dcdf20ef7ba4599b2c2bebcb6113971) J[oseph](https://www.notion.so/Larmarange-Joseph-1dcdf20ef7ba4599b2c2bebcb6113971) \| Centre Population et Développement, Institut de Recherche pour le Développement, Université de Paris, Inserm, Paris, France \| \| [Maheu-Giroux Mathieu](https://www.notion.so/Maheu-Giroux-Mathieu-907fc88b747d4b17b1232b41f16ec5a6) \| Department of Epidemiology, Biostatistics, and Occupational Health, School of Population and Global Health, McGill University, Montréal, QC, H3A 1A2, Canada \| \| Medley Graham \| Department of Global Health and Development, Faculty of Public Health and Policy, London School of Hygiene and Tropical Medicine, London, UK \| \| [Moh Raoul](https://www.notion.so/Moh-Raoul-bd729f67dd37479bb0ff89f3a7e53343) \| Programme PACCI, ANRS Research Site, Treichville University Hospital, Abidjan, Côte d'Ivoire.  Department of Infectious and Tropical Diseases, Treichville University Teaching Hospital, Abidjan, Côte d'Ivoire.  Medical School, University Felix Houphouet Boigny, Abidjan, Côte d'Ivoire \| \| [Ndour Cheikh Tidiane](https://www.notion.so/Pr-Ndour-Cheikh-Tidiane-e66b84ae4d0d495c8aa7bf8c91608ff0) \| Division de Lutte contre le Sida et les IST, Ministère de la Santé et de l'Action Sociale Institut d'Hygiène Sociale, Dakar, Sénégal \| \| [Pourette Dolorès](https://www.notion.so/Pourette-Dolor-s-36402926706145248de586073fc128ce) \| Centre Population et Développement, Institut de Recherche pour le Développement, Université de Paris, Inserm, Paris, France \| \| [Rouveau Nicolas](https://www.notion.so/Rouveau-Nicolas-6ace639301f34853bd4b19dc681aaede) \| Centre Population et Développement, Institut de Recherche pour le Développement, Université de Paris, Inserm, Paris, France \| \| [Silhol Romain](https://www.notion.so/Silhol-Romain-e2fe7bbd45b64ad7a1d354042c4926d2) \| Medical Research Council Centre for Global Infectious Disease Analysis, Department of Infectious Disease Epidemiology, Imperial College London, London, United Kingdom \| \| [Simo Fotso](https://www.notion.so/Arlette-Simo-Fotso-ceee998dafd74b3b935ac9059d4c82a2) Arlette \| Centre Population et Développement, Institut de Recherche pour le Développement, Université de Paris, Inserm, Paris, France \| \| [Terris-Prestholt Fern](https://www.notion.so/Terris-Prestholt-Fern-eae075a74e004b6190a6cbf7d8054e26) \| Department of Global Health and Development, Faculty of Public Health and Policy, London School of Hygiene and Tropical Medicine, London, UK \| \| [Traore](https://www.notion.so/M-togara-Mohamed-Traore-94589a7530a24ed7aefd2d46d1bdf8b1) Métogara Mohamed \| Solthis, Côte d'Ivoire \| \| [Vautier Anthony](https://www.notion.so/Vautier-Anthony-23d8a704ce10434bb8ddf5b4d435ec88) \| Solthis, Dakar, Sénégal \| \| Solthis coordination team \| \| \| Diallo Sanata \| Solthis, Dakar, Sénégal \| \| Gueye Papa Alioune \| Solthis, Dakar, Sénégal \| \| [Geoffroy](https://www.notion.so/Olivier-Geoffroy-9ae6efdb6861466bb292024fe0d3a6d6) [Olivier](https://www.notion.so/Olivier-Geoffroy-9ae6efdb6861466bb292024fe0d3a6d6) \| Solthis, Abidjan, Côte d'Ivoire \| \| Kabemba [Odé Kanku](https://www.notion.so/Od-KANKU-KABEMBA-fdd3be902bd34ac6a17d71323502c7cc) \| Solthis, Bamako, Mali \| \| Implementation in Côte d’Ivoire \| \| \| [Abokon Armand](https://www.notion.so/Dr-Armand-ABOKON-705492b96e56435c801b4282d55a78c8) \| Fondation Ariel Glaser, Côte d'Ivoire \| \| [Anoma Camille](https://www.notion.so/Dr-Camille-ANOMA-384d3874b0ee4a2786eb8c051f325de6) \| Espace Confiance, Côte d'Ivoire \| \| [Diokouri](https://www.notion.so/Dr-Annie-DIOKOURI-9a1e77785b674bfbb4095a7fa7222428) [Annie](https://www.notion.so/Dr-Annie-DIOKOURI-9a1e77785b674bfbb4095a7fa7222428) \| Fondation Ariel Glaser, Côte d'Ivoire \| \| [Kouame Blaise](https://www.notion.so/Dr-Blaise-KOUAME-1e4513a6640f49eeadba700517276986) \| Service Dépistage, PNLS \| \| [Kouakou Venance](https://www.notion.so/Dr-Venance-KOUAKOU-b9c776f673924dd19a8c47e057b55d92) \| Heartland Alliance, Côte d'Ivoire \| \| [Koffi Odette](https://www.notion.so/KOFFI-Odette-d050f4d4459f4076be248a539220f6a6) \| Aprosam, Côte d'Ivoire \| \| Kpolo [Alain-Michel](https://www.notion.so/Alain-Michel-KPOLO-835d3fb5b88a48629613d8dd7338782f) \| Ruban Rouge, Côte d'Ivoire \| \| [Tety Josiane](https://www.notion.so/TETY-Josiane-e46cbc6a44924823ab0e5810c32971b5) \| Blety, Côte d’Ivoire \| \| [Traore Yacouba](https://www.notion.so/TRAORE-Yacouba-f95f844b8db9474893fd229908e5686d) \| ORASUR, Côte d’Ivoire \| \| Implementation in Mali \| \| \| [Bagendabanga Jules](https://www.notion.so/Dr-Jules-Bagendabanga-6095d315dc4343beb4791df9c06ee2ff) \| FHI 360, Mali \| \| Berthé [Djelika](https://www.notion.so/Djelika-Berth-62c2001634444844b89051b0aca8d58b) \| PSI, Mali \| \| Diakite [Daouda](https://www.notion.so/Daouda-Diakite-a85242c23a8d4138b5950096301bf4ac) \| Secrétariat Exécutif du Haut Conseil National de Lutte contre le Sida, Mali \| \| Diakité [Mahamadou](https://www.notion.so/Mahamadou-DIAKITE-1db01b1f327c4db5a5127dd15447a1ae) \| Danayaso, Mali \| \| Diallo [Youssouf](https://www.notion.so/Youssouf-Diallo-86ab4828bb2446ff99be2dca808df663) \| CSLS/MSHP \| \| [Daouda Minta](https://www.notion.so/Prof-Minta-Daouda-bebbe7a5ae9a444296c87a54d625b203) \| Comité scientifique VIH \| \| [Hessou Septime](https://www.notion.so/Dr-Septime-Hessou-65d8bdedbb044d78820f38c7cf13310f) \| Plan Mali \| \| Kanambaye [Saidou](https://www.notion.so/Saidou-Kanambaye-b1b71418cf57498b9c2f9bc600294897) \| PSI, Mali \| \| [Kanoute Abdul Karim](https://www.notion.so/Kanoute-Abdul-Karim-35b65acd9afb4cba9b49f9ec224488fe) \| Plan Mali \| \| [Keita Dembele Bintou](https://www.notion.so/Dr-Dembele-Bintou-Keita-e2e16458a090479a97095c2a9ee99e96) \| Arcad-Sida, Mali \| \| [Koné Dramane](https://www.notion.so/Dr-Dramane-Kon-6377bfbc3e5b4fe39cfbc854011b51d7) \| Secrétariat Exécutif du Haut Conseil National de Lutte contre le Sida, Mali \| \| Koné [Mariam](https://www.notion.so/Mariam-Kon-50ffd9a9bb6a4a949cbe4bb106fd9c27) \| AKS, Mali \| \| [Maiga Almoustapha](https://www.notion.so/Maiga-Almoustapha-8dd26e801081496b825e055c5b189b2c) \| Comité scientifique VIH \| \| [Nouhoum Telly](https://www.notion.so/Dr-Telly-Nouhoum-fb6c2a5b8b05466cad61530e3cabad35) \| CSLS/MSHP \| \| Sanogo [Abdoulaye](https://www.notion.so/Abdoulaye-SANOGO-2e85b004ca4546c19035008bc18e7db6) \| Amprode Sahel, Mali \| \| [Saran Keita Aminata](https://www.notion.so/Dr-KEITA-Aminata-Saran-2eb414403cfe45f3b2677c053ead66fe) \| Soutoura, Mali \| \| Sidibé [Fadiala](https://www.notion.so/Fadiala-Sidib-da56f184c8c44e96b7d0a0ec680da426) \| Soutoura, Mali \| \| Tall [Madani](https://www.notion.so/Madani-Tall-ca7f54d7ebb2481e8dc494cd75059dc0) \| FHI 360, Mali \| \| [Yattassaye Camara Adam](https://www.notion.so/Dr-Camara-Adam-Yattassaye-ab44119c6e34408d9de404f33bec50d1) \| Arcad-Sida, Mali \| \| Implementation in Senegal \| \| \| [Bâ Idrissa](https://www.notion.so/Dr-Idrissa-B-911a7d7b6b3745d59f64344f35717ad8) \| CEPIAD, Sénégal \| \| [Diallo Papa Amadou Niang](https://www.notion.so/Dr-Papa-Amadou-Niang-293bf2ecfc464c3b84df949c71f0d31c) \| CNLS, Sénégal \| \| [Fall Fatou](https://www.notion.so/Dr-Fatou-Fall-7d2d7ed8ae78469eb2efd53b494f0704) \| DLSI, Ministère de la Santé et de l'action sociale, Sénégal \| \| [Guèye NDèye Fatou NGom](https://www.notion.so/Dr-ND-ye-Fatou-NGom-Gu-ye-c3161c72b7d9433eaa1b02cc79a9f8c7) \| CTA, Sénégal \| \| Ndiaye [Sidy Mokhtar](https://www.notion.so/Sidy-Mokhtar-NDiaye-d470d2be528b46fca7779d12cb4ac710) \| Enda Santé, Sénégal \| \| [Niang Alassane Moussa](https://www.notion.so/Dr-Alassane-Moussa-Niang-c52bafcebb69432587c1607a79e0f1d5) \| DLSI, Ministère de la Santé et de l'action sociale, Sénégal \| \| [Samba Oumar](https://www.notion.so/Dr-Oumar-Samba-df3d5f1bb0e641e5803e39a008b8cf64) \| CEPIAD, Sénégal \| \| [Thiam Safiatou](https://www.notion.so/Dr-Safiatou-Thiam-fbca1b25bd7b4f29baa0c7bbf08b03e9) \| CNLS, Sénégal \| \| Turpin [Nguissali M.E.](https://www.notion.so/Nguissali-M-E-Turpin-3fe470426b784b56ad7dd877b40bbc8b) \| Enda Santé, Sénégal \| \| Partners \| \| \| Bouaré [Seydou](https://www.notion.so/Seydou-Bouar-f3669d3cfacd4b77ab99da4cad9c7934) \| Assistant de recherche, Mali \| \| [Camara Cheick Sidi](https://www.notion.so/Cheick-Sidi-Camara-dda932a72bb046568190b9b5cb3e30c0) \| Assistant de recherche, Mali \| \| Eponon Ehua Agnes \| Assistante de recherche, Sénégal \| \| Kouvahe Amélé \| Stagiaire IRD \| \| Montaufray Marie-Anne \| Stagiaire IRD \| \| [Mosso Rosine](https://www.notion.so/Mosso-Rosine-12dade63b5d2418c9ddb4cec4570dc80) \| ENSEA Ecole Nationale de Statistiques et d'Economie Appliquée, Abidjan, Côte d'Ivoire \| \| Ndeye Pauline Dama \| Chargée de l’enquête coupons, Sénégal \| \| [Sarrassat Sophie](https://www.notion.so/Sarrassat-Sophie-c197183699454536a53ef3ad56c4cc82) \| Centre for Maternal, Adolescent, Reproductive and Child Health, London School of Hygiene and Tropical Medicine, London, UK \| \| Sow [Souleymane](https://www.notion.so/Souleymane-Sow-6ca1b6d006d6497a96d1e72b050824c7) \| Assistant de recherche, Sénégal \| |
| --- | --- | --- | --- | --- | --- | --- | --- | --- | --- | --- | --- | --- | --- | --- | --- | --- | --- | --- | --- | --- | --- | --- | --- | --- | --- | --- | --- | --- | --- | --- | --- | --- | --- | --- | --- | --- | --- | --- | --- | --- | --- | --- | --- | --- | --- | --- | --- | --- | --- | --- | --- | --- | --- | --- | --- | --- | --- | --- | --- | --- | --- | --- | --- | --- | --- | --- | --- | --- | --- | --- | --- | --- | --- | --- | --- | --- | --- | --- | --- | --- | --- | --- | --- | --- | --- | --- | --- | --- | --- | --- | --- | --- | --- | --- | --- | --- | --- | --- | --- | --- | --- | --- | --- | --- | --- | --- | --- | --- | --- | --- | --- | --- | --- | --- | --- | --- | --- | --- | --- | --- | --- | --- | --- | --- | --- | --- | --- | --- | --- | --- | --- | --- | --- | --- | --- | --- | --- | --- | --- | --- | --- | --- | --- | --- | --- | --- | --- | --- | --- | --- | --- | --- | --- | --- | --- | --- | --- | --- | --- | --- | --- | --- | --- | --- | --- | --- | --- | --- | --- | --- | --- | --- | --- | --- |

## Data and R code

Data and R codes supporting the results of the paper are provided as supplementary material. See the provided supplementary files (see files data.csv and analysis.Rmd).
